# Healthy Eating and Active Lifestyle After Bowel Cancer (HEAL ABC): feasibility randomised controlled trial protocol

**DOI:** 10.1186/s40814-020-00721-y

**Published:** 2020-11-13

**Authors:** Jana Sremanakova, Anne Marie Sowerbutts, Chris Todd, Richard Cooke, Sorrel Burden

**Affiliations:** 1 School of Health Sciences, University of Manchester, Oxford Road, Manchester, M13 9PL UK; 2 Manchester Academic Health Science Centre, Manchester, UK; 3 Manchester University NHS Foundation Trust, Manchester, UK; 4 NIHR Applied Research Collaboration Greater Manchester, Manchester, UK; 5 Department of Psychology, University of Liverpool, Liverpool, UK; 6 Salford Royal NHS Foundation Trust, Salford, UK

**Keywords:** Colorectal, Cancer, Diet, Physical activity, Behaviour change, Survivorship

## Abstract

**Background:**

Targeting modifiable lifestyle factors including diet and physical activity represents a potentially cost-effective strategy that could support a growing population of colorectal cancer survivors and improve their health outcomes. Currently, effective, evidence-based interventions and resources helping people after bowel cancer to adopt new lifestyle habits are lacking. The aim of this trial is to test the Healthy Eating and Active Lifestyle After Bowel Cancer (HEAL-ABC) intervention to inform a future definitive randomised controlled trial.

**Methods/design:**

This is a feasibility randomised controlled trial. A total of 72 survivors who have completed surgery and all anticancer treatments will be recruited. The intervention group will receive HEAL-ABC resources based on behaviour change theory combined with supportive telephone calls informed by motivational interviewing every 2 weeks during the 3-month intervention, and once a month for 6 months to follow-up. Participants in the control group will follow usual care and have access to resources available in the public domain. The study is testing feasibility of the intervention including adherence and ability to collect data on anthropometry, body composition, diet, physical activity, behaviour change, quality of life, blood markers, contact with healthcare services, morbidities and overall survival.

**Discussion:**

The proposed study will add to the evidence base by addressing an area where there is a paucity of data. This study on lifestyle interventions for people after colorectal cancer follows the Medical Research Council guidance on evaluating complex interventions in clinical practice. It focuses on people living after treatment for colorectal cancer and targets an important research area identified by cancer survivors as a research priority reported by the National Cancer Institute and James Lind Alliance UK.

**Trial registration:**

ClinicalTrials.gov NCT04227353 approved on the 13th of January 2020

**Supplementary Information:**

The online version contains supplementary material available at 10.1186/s40814-020-00721-y.

## Background

Over recent decades, the number of people living with and beyond cancer has continued to rise worldwide. Current survival rates are associated with increased cancer incidence reaching 18 million new cases in 2018 [1], ageing of the population [2] and advances in early cancer detection and treatment [3]. Several definitions of cancer survivorship have been proposed. One approach in research and within the medical community defines a person who has survived cancer as “living with and beyond cancer” [4].

There is mounting evidence that links lifestyle choices regarding diet and physical activity to primary cancer risk [5]. Likewise, large cohort studies have shown that people who have survived cancer and followed healthy eating and active lifestyles can improve their survival rate [6]. It is therefore hypothesised that adherence to healthy diet and physical activity in people who have completed cancer treatment may reduce cancer risk, recurrences, comorbidities, new cancers, cardiovascular diseases and diabetes and improve overall survival rates [7]. However, there is insufficient evidence from randomised controlled trials (RCTs) to support this hypothesis. This has been highlighted in a systematic review on dietary interventions in adult cancer survivors, which revealed an uneven distribution of research across cancer sites [8].

Currently, evidence in cancer survivorship is centred predominantly on breast cancer studies [8, 9]. There is a paucity of evidence for colorectal, gynaecological and prostate cancer. The global burden of colorectal cancer (CRC) is expected to increase by 60%, which is more than 2.2 million new cases, by 2030 [10]. In England and Wales, almost six out of every ten people diagnosed with CRC survive their disease for 10 years or more [11]. However, survival is often affected by a number of physical and psychological problems including comorbidities [12], side effects from treatment including neuropathy, bowel disturbances [13], weight changes [14], cancer recurrences [15] or new cancer diagnosis [16] and surgical procedures can leave a proportion of patients with stoma. All these factors have a substantial impact on survivors’ quality of life [17, 18]. Helping people after cancer to follow a healthier lifestyle is a low-cost strategy that has the potential to mitigate health complications people may face after their treatment. To date, evidence indicates limited success at initiating long-term lifestyle changes in people living after cancer [8, 19] and effective evidence-based approaches to help people make substantial lifestyle changes after CRC are lacking.

Difficulties in identifying an effective lifestyle intervention may be influenced by the variety of interventions that have been tested and also by limitations in resources allocated to the development of an appropriate intervention. Dietary interventions are complex; however, there is guidance available from the UK Medical Research Council (MRC) on the development and evaluation of complex interventions in healthcare [20]. This guidance suggests piloting and feasibility testing to enhance the development and integration of complex interventions into healthcare. It also suggests involvement of participants in intervention development and conducting qualitative work to test acceptability, barriers and facilitators to adherence and uptake. There are diet and lifestyle interventions that have been developed incorporating some of these points to a high standard for people after breast cancer [21–23]; however, high-quality interventions for people after CRC are currently absent.

The proposed study aims to build on the current knowledge base by following MRC Guidance [20]. Prior to the protocol development, people after CRC were asked about their views and experiences of eating after a cancer diagnosis and their motivation for change [18] along with their preferences regarding delivery of lifestyle information [24]. This was combined with the development of an intervention resources which involved work with CRC survivors’ and healthcare professionals [25]. Behaviour change theory was integrated throughout the resource [26].

The purpose of this study is to evaluate the feasibility of conducting a fully powered trial for a Healthy Eating and Active Lifestyle After Bowel Cancer (HEAL ABC) intervention versus usual care.

## Methods/design

This is a feasibility parallel group RCT using 1:1 randomisation*.* The study follows CONSORT guidelines for reporting a pilot or feasibility trial [27] and follows the Standard Protocol Items: Recommendations for Interventional Trials (SPIRIT) [28]. Version 6 of the protocol has been finalised on 7th of January 2020.

### Eligibility and exclusion

Participants will be included in the trial if they are over 18 years old, more than 12 weeks post-surgery and have completed all active anti-cancer treatments (surgery, radiotherapy or chemotherapy). Detailed inclusion and exclusion criteria are presented in Table 1.

**Table 1:** Inclusion and exclusion criteria

Inclusion criteria	Exclusion criteria
Adults, age ≥ 18	Age < 18
Minimum 12 weeks post-surgery and/or active treatment	Less than 12 weeks post-surgery or active treatment
Completed all active anti-cancer treatments, including surgery, radiotherapy or chemotherapy	Receiving treatment for malignancy, secondary malignancy
No serious health complications	Short bowel syndrome, Crohn’s disease, ulcerative colitis, diverticulitis or jejunostomy, previous stroke, congested cardiac failure or oedema, hepatic or renal failure
No specific dietary requirements	On any therapeutic diets, multiple food intolerances or allergies
Body Mass Index ≥ 20 kg/m^2^	Body Mass Index < 20 kg/m^2^
Unintentional weight loss ≤ 5% in the previous 3–6 months	Unintentional weight loss >5% in the previous 3–6 months
Following < 4 of the WCRF/AICR recommendations	Already following ≥ 4 WCRF/AICR recommendations
Ability to work with computer, smart phone or tablet	Inability to work with computer, smart phone or tablet
Ability to give informed consent	Inability to give informed consent

*WCRF/AICR* World cancer Research Fund and American Institute of Cancer Research

### Recruitment and consent

**Fig. 1:**
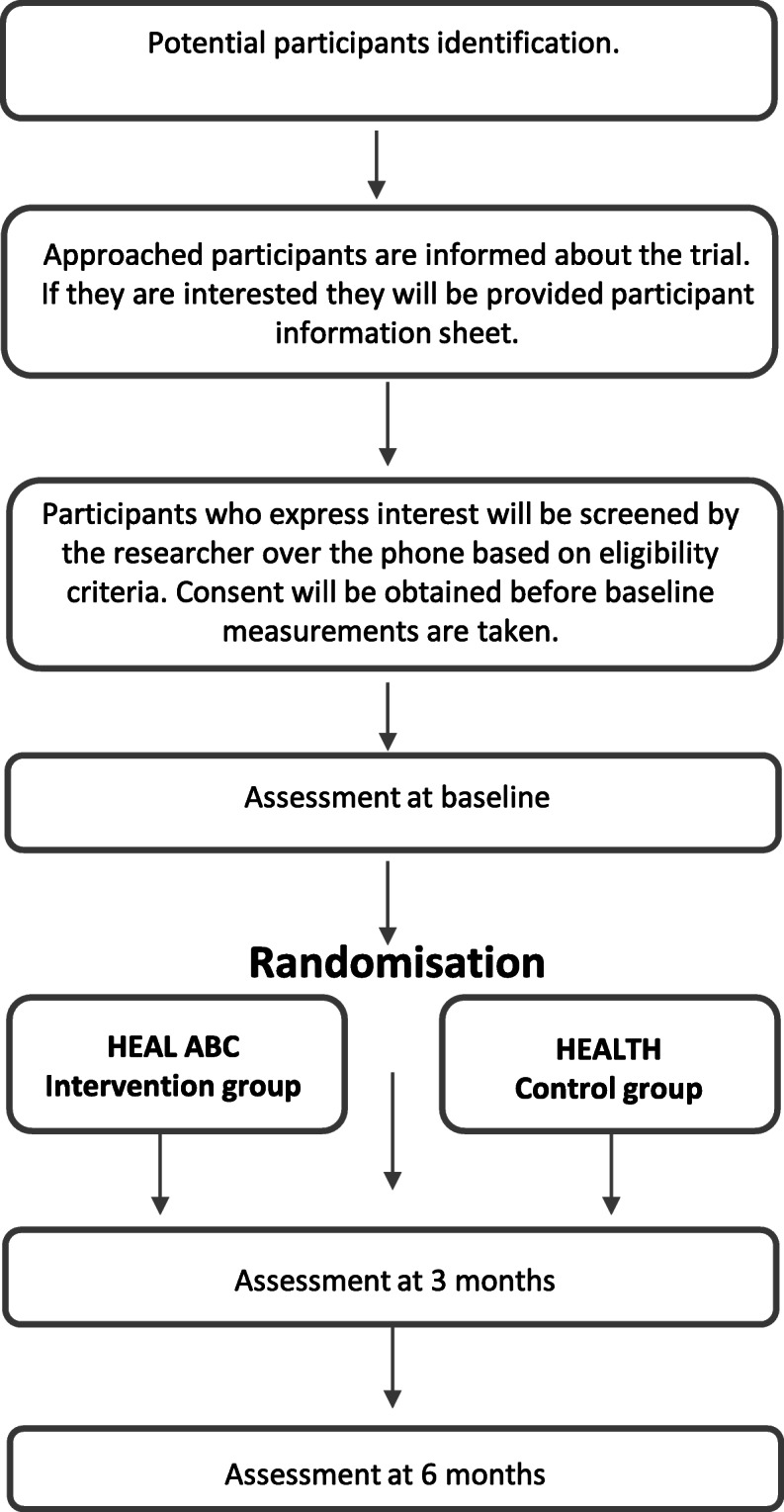
Study flow diagram

Participants will be recruited from outpatient CRC surveillance clinics in Great Manchester (Fig. 1). In the hospitals, appropriate participants will be identified from clinical records and screened by clinic staff. A record of people approached and individuals that express an interest will be kept. A patient information sheet and researcher contact details will be provided to facilitate follow up of potentially interested participants. The COVID-19 pandemic in UK in 2020 may preclude using this face to face approach, in which case an alternative non-face to face method of recruitment will be adopted. Participants who are interested in taking part will be consented, randomised and allocated to one of two trial arms. Baseline data will be collected following randomisation to the control or intervention arms.

### Intervention

**Fig. 2:**
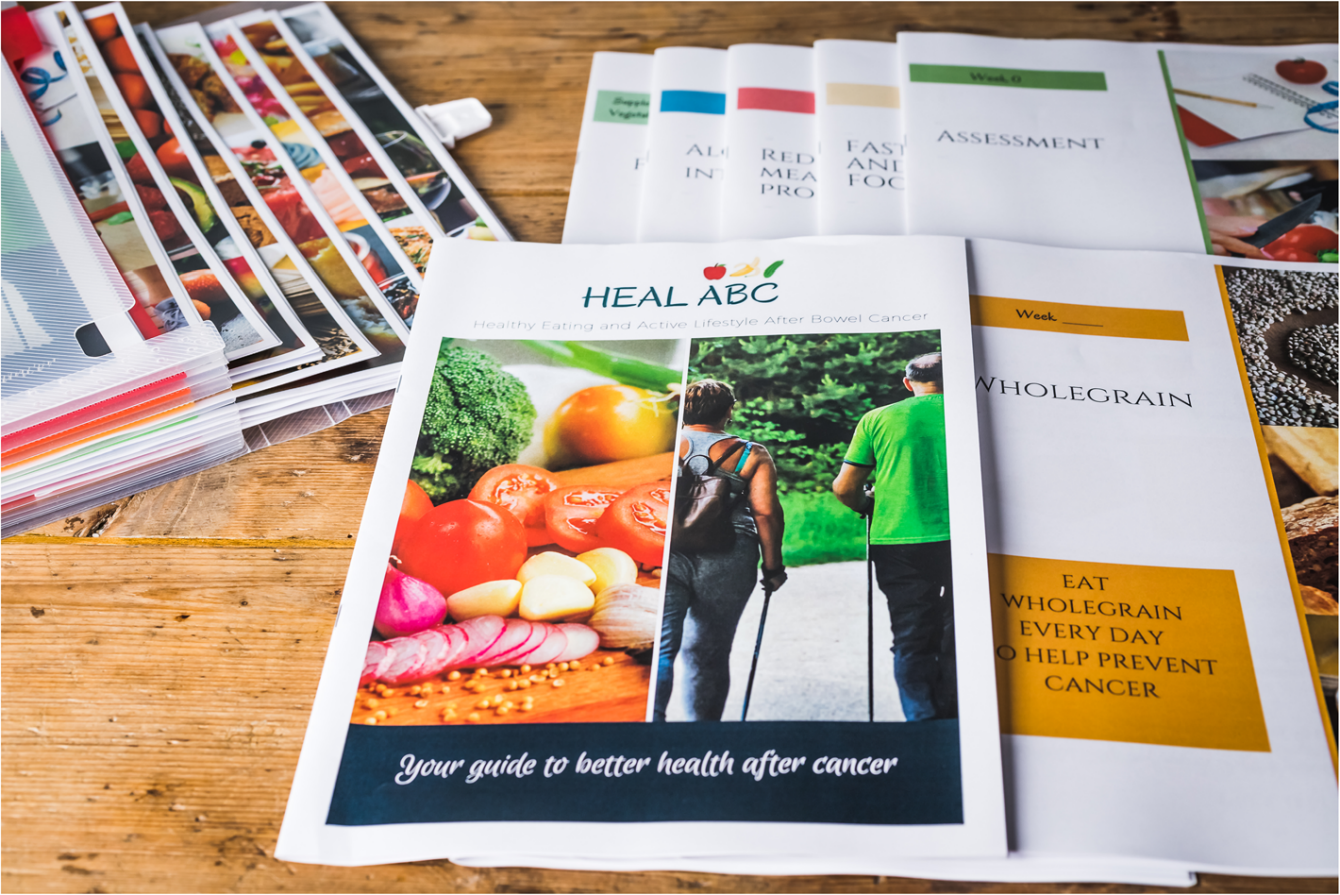
Healthy Eating and Active Lifestyle After Bowel Cancer—HEAL ABC resources

The intervention group will follow the HEAL ABC programme involving a written resource (Fig. 2) combined with supportive telephone calls every 2 weeks during the intervention and once a month during the follow-up period.

### HEAL ABC resources

**Fig. 3:**
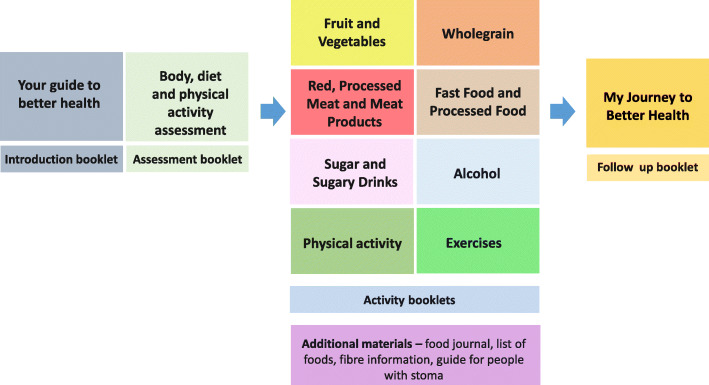
Healthy Eating and Active Lifestyle After Bowel Cancer—HEAL ABC structure [25]

Participants allocated to the intervention will be encouraged to follow HEAL ABC resources (Fig. 3) in order to make a healthy eating and active lifestyle changes. Development of the HEAL ABC resources and qualitative evaluation from people after bowel cancer, healthcare professionals and researchers is described elsewhere [25].

Resources are based on the World Cancer Research Fund and American Institute of Cancer Research (WCRF/AICR) guidelines on diet and physical activity for cancer prevention [7, 29]. Suggestions for physical activity and exercise are based on a strategy, which integrates physical activity into everyday life tasks, that has been shown to be more effective than structured exercise programmes in exercise facilities [30, 31]. Resources have incorporated recommendations from the Health Action Process Approach (HAPA) [26], a behaviour change theory.

### Behaviour change

The HEAL ABC resources consist of 12 booklets and additional supportive sheets. The order of the booklets is not specified. Participants start with an assessment booklet and review their current diet and physical activity level. Following the assessment, participants will be assigned their first booklet based on the one they perceive to be the easiest for setting an incremental goal and achieving it [32]. Meaning participants start with the booklet of the highest self-efficacy and after they achieve a successful behaviour change, they can move more confidently to the next booklet. During the follow-up period, participants will use the follow up booklet, which helps them to continue with changes they have made and encourage them to set new goals (Table 2).

**Table 2:** Content, strategies and behavioural targets of the Healthy Eating and Active Lifestyle After Bowel Cancer programme

Target	Task	Source
Increase awareness of the need to change	Assessment of body, diet and physical activity	Assessment booklet
Understanding benefits of healthy eating habits and activity lifestyle	Information about healthy recommendations	All booklets
Learning about new skills on healthy eating and active lifestyle	Learning about healthy options, cooking and shopping tips, understanding portion sizes, choosing healthier alternatives/adding activities to daily routines	All diet-related booklets/physical activity booklet
Exploring current habits and identifying where the change can be made	Targeted questions on specific dietary habits/physical activity	All diet-related booklets/physical activity booklet
Selections of specific goal(s)	Action plan	All booklets
Creating detailed plan how to achieve selected goal	Action plan	All booklets
Understanding own motivation, risks, barriers, coping strategies and readiness to change	Get ready for your action section	All booklets
Calendar with notes to record	Make your notes section	All booklets
Review of the goal and action	Review plan	All booklets
Instruction to start new booklet, follow-up with participants progress, encouragement and support	Supportive calls every 2 weeks during intervention and once a month during follow up	Telephone calls
Maintenance of new behaviour, encouragement to start new changes	Calendar for 12 weeks to track progress and continue with changes, opportunity to plan and track new goals	My journey to better health booklet

The HAPA model for behaviour change was selected for the resources as it has been developed directly to target health-related behaviours [26]. This theory takes into consideration behavioural aspects that support the formation of intention to change to post-intentional phase of behaviour maintenance and recovery. The HAPA constructs have been mapped in HEAL ABC booklets, incorporated into tasks and activities within the booklets to support intention, action, maintenance and the recovery self-efficacy of study participants (Table 3).

**Table 3:** Health Action Process Approach (HAPA) constructs mapped within the Healthy Eating and Active Lifestyle After Bowel programme

Construct	Implementation in HEAL ABC programme	Construct's description
Action self-efficacy	Get ready page in the booklet—how confident are you about making this change?	Participants believe in their own ability to improve their diet and to become more active.
Outcome expectancies	Get ready page in the booklet—why do you want to make this change?	Participants believe they will benefit from changing their behaviour and achieve positive outcomes when taking part in the HEAL ABC programme.
Risk perception	Get ready page in the booklet—why do you want to make this change?	Participants believe there will be negative consequences if they do not change their dietary habits and activity level.
Action planning	Action plan page in the booklet—creating a specific action plan	Participants are supported to identify opportunities to incorporate new eating habit(s) and activity(s) into their everyday lives.
Coping planning	Get ready page in the booklet—if things do not go as you plan, what steps will you take to make sure you stick to your plan?	Participants are supported to identify barriers and make plans to address them.
Coping self-efficacy	Motivational interviewing during the phone calls	Participants believe in their capacity to continue with a new behaviour even when barriers arise.
Recovery self-efficacy	Motivational interviewing during the phone calls	Participants believe they can return to their new behaviour even when they disengaged with their new activities for a long period.

### Telephone calls during 3-month and 6-month periods

Participants in the intervention group will receive a telephone call every 2 weeks during the intervention period and once a month during the follow-up period. Calls aim to support participants’ use of HEAL ABC resources to facilitate behaviour change by reviewing participants’ goals for desired behaviour change. The structure of the telephone calls will be informed by motivational interviewing [33], with the interviewer trained in motivation interviewing technique. The interviewer will explore participant’s own ability to identify the most appropriate plan for their action and explore their strengths and capabilities to change. Guidance will be provided to participants in order to set new goals if satisfactory changes were initiated. The telephone calls will be audio-recorded with an encrypted voice recorder to enable the monitoring and reviewing of the calls and evaluation of the intervention fidelity [34].

### Control (HEALTH group)

Participants allocated to the control group will be referred to the healthy lifestyle recommendations available at the World Cancer Research Fund, Bowel Cancer UK and UK government websites. No additional support or telephone calls will be provided.

### Assessment and outcomes

Both intervention and control group are assessed at baseline, 3- and 6-month data collection points. The schedule of enrolment, interventions and assessments is presented in Table 4.

**Table 4:** Schedule for measurement, testing and assessment at baseline and follow-up assessment

Measure/assessment	Method of assessment	Prior baseline	Baseline	Post intervention 3 months	Follow-up 6 months
Enrolment	-	✓	x	x	x
Eligibility screen	-	✓	x	x	x
Informed consent	-	✓	x	x	x
Allocation	-	x	✓	x	x
Participants’ characteristics	Sociodemographic questionnaire	x	✓	x	x
Patients’ medical history	Medical records	x	✓	x	x
Anthropometry	Scale, tape measure, stadiometer	x	✓	✓	✓
Body composition	BIA, CT scans analysis	x	✓	✓	✓
Dietary assessment	3-day food diary, 24 dietary recall	x	✓	✓	✓
Physical activity level	GPAQ Questionnaire	x	✓	✓	✓
Step count	Pedometer	x	✓	✓	✓
Behaviour/motivation	HAPA questionnaire	x	✓	✓	✓
Participant’s experience	In-depth interviews	x	x	✓	✓
Quality of life	SF-12 questionnaire	x	✓	✓	✓
Participants’ contact with healthcare services	Healthcare Resource Use Questionnaire	x	x	x	✓
Morbidities, mortality	Medical records	x	x	x	✓

*BIA* bio-impedance analysis, *CT* computer tomography, *GPAQ* Global Physical Activity Questionnaire, *SF-12* Short form 12 quality of life

### Baseline assessment

At baseline, details of patients’ characteristics will be collected using a sociodemographic questionnaire (see Additional file 1). Patient medical records will be used to collect information on cancer staging using the Classification of Malignant Tumours (TNM classification) post-surgery [35], type of chemotherapy/radiotherapy, disease site and operation details.

Further baseline measurements will include anthropometry, body composition, dietary intake, physical activity, quality of life, behaviour change assessment and blood tests.

#### Anthropometry and body composition

Standard operating procedures will be used to ensure consistency of the measurements. Height will be measured using a stadiometer and records rounded to the nearest centimetre (cm) (Harpenden pocket stadiometer Practical Metrology, Sussex, UK). Body weight will be recorded to the nearest 0.1 kg. Body composition will be measured using bioelectrical impedance analysis (DC-430 MA, Tanita Europe BV, the Netherlands) to assess fat mass and fat-free mass. We will collect copies of computer tomography (CT) scans for participants taken at the closest date to our assessment. The CT scans will be analysed using Slice-o-Matic (Tomovision 5.0, Canada) and Image J (ImageJ, US National Institutes of Health, Bethesda, MA, USA) software. Single axial images at the level of the third lumbar vertebrae will be used to measure total skeletal muscle and total fat mass at the cross-sectional area. Hounsfield units (HU) threshold will be set at − 190 to − 30 for subcutaneous fat, − 150 to − 50 for visceral fat, − 190 to − 30 for intramuscular fat and − 29 to 150 for skeletal muscle. The skeletal muscle area will be normalised for stature by calculating the skeletal muscle index. Muscle mass and fat mass will be calculated using standard equations [36].

#### Dietary assessment

Diet will be assessed using a 3-day food diary and electronic dietary recall. Participants will complete a food diary prospectively during the day and enter all foods and drinks consumed into an online system for dietary assessment, INTAKE24 [37], at the end of each day. INTAKE24 is a self-completed online 24-h dietary recall compliant with the general data protection regulation (2018). To complete the dietary recall, participants will be assigned a specific number, which they receive by email. This will serve as a log enabling access to the online dietary record. Participants will be instructed how to complete a food diary and INTAKE24 recall by the researcher, who is a qualified nutritionist. Data collected will be used to assess energy and nutrient consumption, as well as, frequency of fruit and vegetables, red meat, processed meat, fast food, sugary drinks, sweets, and alcohol. This method of food frequency assessment will replace a standard food frequency questionnaire, which is subjected to reporting error due to the inaccuracy of absolute nutrient values, variation of nutrient values depending on questionnaire length and structure and lack of details recorded about food consumed [38, 39]. Further advantages of combining food diary and electronic recall relate to participant’s age and memory capacity, as the average age of CRC survivors is above 65 years. In addition, a Diet Quality Index will be calculated based on data collected [40].

#### Physical activity level

The Global Physical Activity Questionnaire (GPAQ) will be used to assess participant’s level of physical activity [41]. A validated pedometer will be used [42, 43] to measures daily steps for a week at each time point. Participants will be instructed to wear the pedometer during the day clipped to their waistband on either the left or right hip.

#### Quality of life

Health-related quality of life will be assessed using the SF-12 questionnaire [44], selected for its validity, generalisability and simplicity [45, 46].

#### Behaviour change

Behaviour change will be assessed using a behaviour change questionnaire, using questions derived from the HAPA. This questionnaire was developed to explore changes in different HAPA constructs (aspects related to participant’s behaviour). The HAPA questionnaire has been previously used to investigate changes in HAPA constructs in lifestyle interventions [47–49].

#### Biochemistry and haematology

Blood test results of routinely collected inflammatory markers (C-reactive protein, leukocytes level, albumin, haemoglobin) will be collected from patient’s medical records [50].

### Post-intervention assessment at 3 months

At 3 months, all baseline measurements will be repeated. In addition, a subset of 12 to 15 participants in the intervention group will be interviewed using qualitative interviews. The interviews will aim to gain an insight into participants’ experience of being in the study, using HEAL ABC resources, and will explore motivations, barriers and facilitators regarding adherence to the intervention. A subset of 12 to 15 participants in the control group will also be interviewed about the experience in the study, their level of motivation and action taken towards a healthier lifestyle.

### Follow-up assessment at 6 months

At 6 months, all baseline measurements will be repeated, and qualitative interviews will be performed again with the same subset of participants. Additionally, cancer recurrences, morbidities and survival will be collected from patients’ medical records and health resource questionnaire provided.

#### Healthcare Resource Use Questionnaire

A health economics questionnaire will be used to find out about patient’s contact with primary and secondary healthcare services. The questionnaire was developed by a health economist for the European Union funded project “PreventIT” [51].

### Qualitative interviews at 3 and 6 months

We will aim to recruit participants for interviews until data saturation is achieved and we anticipate this will be between 12 and 15 participants [52]. Overall, this will provide 24–30 interviews in total, and 12–15 interviews at each time point is considered to be sufficient to reach data saturation as recommended by Guest et al. [53]. Participants will be selected at baseline, as the first 12 to 15 who agree to be interviewed. This will allow an early evaluation of participants’ experience of the study. A topic guide developed for the study will be used to conduct the interviews. The guide will cover topics such as experience of randomisation, intervention, HEAL ABC resources and data collection. It will also discuss motivation, barriers and facilitators for following the HEAL ABC intervention. The topic guide will be continually reviewed throughout the interview process to ensure it covers any emerging topics of interest. Interviews will be audio-recorded using an encrypted audio recorder and will be fully transcribed verbatim.

### Outcomes

Outcome related to feasibility will be assessed by adherence to WCRF/AICR guidelines, recruitment rates, retention rates, data completion rates and loss to follow up. Adherence to intervention will be assessed as adherence to the WCRF/AICR guidelines by using a scoring system for nutrition and physical activity guideline adherence developed by McCullough and colleagues [54, 55]. We will also assess number of goals set and number of changes implemented in everyday life during the 3-month intervention. Recruitment rates will be assessed as a cumulative recruitment against target rate each month and retention rates calculated as the number of participants who completed the study divided by the number of participants randomised. Acceptability of the intervention will be explored qualitatively through interviews with a subset of the study participants. Data completion rates will be judged as percentage of missing data and completeness of data for all outcome measures at all the time points. Changes in means and standard deviation will be compared to allow for the sample size calculation for a future trial.

Other outcomes include assessing the practicality of data collection for dietary intake, physical activity levels, behaviour change in relation to diet and physical activity and determining effect sizes for these outcomes. Interviews will be performed to explore participants’ experience, motivation, barriers and facilitators to use HEAL ABC resources and follow the intervention.

In the future, a fully powered RCT will test the efficacy of the intervention alongside outcomes collected in the feasibility trial.

### Sample size

We plan to complete the study with 60 participants allowing for up to 15% drop out over 12 months so will recruit a total of 72 participants. This is based on drop out data identified in a systematic review of the literature [8]. As this study does not aim to test a hypothesis, we follow recommendations for feasibility studies by Lancaster et al. [56].

### Randomisation

Participants will be randomised into control and intervention groups using the sealedenvelope.com [57] block randomisation. The randomisation will be stratified for hospital site and cancer site (colon or rectum). An independent person outside of the research team will generate the allocation sequence and assign participants into their groups. Participants cannot be completely blinded to the intervention but effort was taken to blind differences between HEAL and HEALTH group for participants through the provision of written materials and following the same assessment. The researcher will not be blinded to the intervention and outcomes.

### Statistical analysis

Quantitative data will be analysed using descriptive statistics displayed with 95% confidence intervals. Changes in outcomes between the intervention arm and control, estimate parameters using means and standard deviations will be descriptively compared and will inform a sample size calculation for a full RCT. All quantitative data will be analysed in STATA 15 (StataCorp, TX: StataCorp LLC) [58].

Qualitative interview transcripts will be managed using NVivo 12 software (QSR International Pty Ltd., Doncaster, VIC, Australia) and analysed using the five stages of framework analysis [59]: familiarisation, developing a thematic framework, indexing, charting and mapping and interpretation. During the first stage (familiarisation) the researcher will become immersed in the data, by reading and re-reading the transcripts. Next, a thematic framework will be developed. After this, transcripts will be indexed (coded) line by line using the thematic framework but remaining open to new themes that emerge. Next, the data will be entered into a chart, so that coded extracts can be attributed to individual participants. Finally, participants’ views will be compared and contrasted, and the data presented schematically (mapping). Contrasting explanations will be explored.

## Discussion

Cancer survivorship research is a relatively new research area but has been identified as addressing the important gap in cancer research [7]. Other researchers have been pioneers in cancer survivorship research emphasising the promotion of long-term health for cancer survivors [60], developed an evidence based interventions primarily for breast cancer survivors [61, 62] and demonstrated positive changes in dietary and other health outcomes in several RCTs [8, 21, 23]. In colorectal cancer, only a few trials have been published to date [63, 64], and many gaps exist within the evidence base [8]; therefore, more research is required in this cancer population.

In recent years, a few study protocols have been published on lifestyle interventions for people after cancer. The Advancing Survival after Cancer Outcomes Trial (ASCOT) study has been designed for all cancer survivors [65]. However, one could argue that there are fundamental differences in diet and physical activity requirements between people with different cancers. It is therefore difficult to apply general lifestyle interventions equally to those after breast cancer and those after CRC, due to specific consequences of treatment including the presence of a stoma. Thus, more tailored interventions and resources addressing needs of specific survivor populations seem necessary. Furthermore, the Norwegian dietary guidelines and colorectal cancer survival (CRC-NORDIET) protocol has been designed as a very opportunistic intervention that offers participants healthy food delivery, cooking classes, individual consultations with a dietitian, access to a training studio and other benefits [66]. This trial with a 14-year follow-up period has a great potential to demonstrate that healthy eating and active lifestyles have a positive impact on health outcomes of people after CRC and their overall survival. However, such an intervention is very unlikely to be accepted by public health policy commissioners and translated into patient care pathways due to high levels of resources required to sustain and unrealistic demands on service providers within healthcare.

The proposed RCT aims to evaluate the feasibility of conducting a fully powered trial for the HEAL ABC intervention. This study has been developed from previous research within the area of CRC survivorship including qualitative work [18, 25], discrete choice experiment [24] and systematic reviews [19, 63]. The proposed study attempts to address areas where research is lacking in the evidence base identified by a Cochrane Systematic review on dietary interventions for cancer survivors [8]. It is based on extensive qualitative work with CRC survivors, which is in line with evidence published by other research groups [17, 67–70]. In conjunction, the intervention has incorporated behaviour change theory and the study aims to test the implementation of this behaviour theory during delivery of the intervention.

### Study limitations

The study is a multicentre feasibility RCT involving hospitals across Greater Manchester and thus the results might not be completely generalisable to the whole of the UK or other countries. The assessment process for most of the study outcomes is based on questionnaires, dietary records and audio recordings. Hence, the research relies heavily on participants’ recall and accurate reporting.

### Summary

To date, many trials have focused on the efficacy of the intervention and overlooked important methodological steps in the research design that might impact on adherence to the intervention and behaviour change. If the intervention is not successful in achieving a high level of adherence and participants do not change their behaviour, there is limited potential to observe any differences between the groups for nutritional, clinical and behavioural outcomes. Hence, improvement in design, study resources and conduct of clinical trials are imperative and possibly can improve the efficacy of evaluating lifestyle interventions designed for survivors of cancer.

## Supplementary Information

**Additional file 1.** Sociodemographic questionnaire.

**Additional file 2.** SPIRIT checklist.

## Data Availability

Not applicable.

## Abbreviations

BIA: Bio-impedance analysis
CRC: Colorectal cancer
CT: Computer tomography
HAPA: Health Action Process Approach
HEAL ABC: Healthy Eating and Active Lifestyle After Bowel Cancer
GPAQ: Global Physical Activity Questionnaire
MRC: Medical Research Council
RCTs: Randomised controlled trials
SF-12: Short form 12 quality of life questionnaire
WCRF/AICR: World Cancer Research Fund and American Institute of Cancer Research
