# Supplementary material for: Healthy Eating and Active Lifestyle After Bowel Cancer (HEAL ABC): feasibility randomised controlled trial protocol
Source: Pilot Feasibility Stud. 2020 Nov 13;6:176. doi: 10.1186/s40814-020-00721-y (PMC7661321; doi:10.1186/s40814-020-00721-y)
Supplement: Supplementary file 1 — Additional file 1. Sociodemographic questionnaire. [file 40814_2020_721_MOESM1_ESM.pdf]

Sociodemographic questionnaire

Participant's number: ☐

Study title: Healthy Eating and Active Lifestyle After Bowel Cancer

We would now like you to complete these questions that tell us about your background. These details will be kept confidential. If you do not wish to answer some of these questions you do not have to.

Background Information

Please answer each question or tick the relevant box.

1. What is your age? *(Please add your age in years)*

☐ Years

2. What is your gender? *(Please tick one of the boxes)*

Female ☐ Male ☐ Transgender ☐ Prefer not to say ☐

3. What is your marital status? *(Please tick one of the boxes)*

Single ☐ Living with partner ☐ Married ☐
Separated/divorced ☐ Widowed ☐

4. What is your occupation (previous occupation if retired)?
(Please write on the line)

----

5. What is the highest level of education you achieve?
(Please tick one of the boxes)

No formal qualifications ☐
Trade qualification or NVQ ☐
GCSE level/or equivalent ☐
A Level/or equivalent ☐
Higher Education Diploma ☐
Degree/or equivalent ☐
Higher degree (MSc/PhD) ☐

6. **What is your ethnic origin?** *(Please tick one of the boxes)*

British	☐	**Black or black British**	
Irish	☐	Caribbean	☐
Other	☐	African	☐
**Mixed**		Other	☐
White & Black Caribbean	☐	**Asian or Asian British**	
White & Black African	☐	Indian	☐
White & Asian	☐	Pakistani	☐
Other	☐	Bangladeshi	☐
**Chinese**		Other	☐
Chinese	☐	**Other Ethnic Group**	
		Other	☐

7. **What is the total income coming into your household each month?**
(Please tick one of the boxes)

Under £250 ☐ £251 to 500 ☐ £501 to 1000 ☐

£1001 to 2000 ☐ over £2000 ☐ Do not want to answer this question ☐

8. **What are the first 3 or 4 digits of your post code?**
(Please write in the boxes)

☐ ☐ ☐ ☐

9. **What is your smoking status?**
(Please tick one of the boxes)

Never smoked ☐ Ex-smoker ☐ Current smoker ☐

10. **What supplements (e.g. vitamin D, iron, herbal supplements) do you use and how often?**
(Please write on the lines)

____

____

____

____

____

Thank you very much for your answers.
